# The impact of subtotal pancreatectomy on people with congenital hyperinsulinism and their caregivers

**DOI:** 10.3389/fendo.2026.1787064

**Published:** 2026-06-22

**Authors:** Kristen E. Rohli, Indraneel Banerjee, Henrik Thybo Christesen, Diva D. De Leon, Lauren N. Lopez, Julie Raskin, Tai L. S. Pasquini

**Affiliations:** 1 Congenital Hyperinsulinism International, Glen Ridge, NJ, United States; 2 Department of Paediatric Endocrinology, Royal Manchester Children’s Hospital, Manchester, United Kingdom; 3 Department of Clinical Research Faculty of Health Sciences, University of Southern Denmark, Odense, Denmark; 4 Hans Christian Andersen Children’s Hospital, Odense University Hospital, Odense, Denmark; 5 Congenital Hyperinsulinism Center and Division of Endocrinology and Diabetes, Children’s Hospital of Philadelphia, Philadelphia, PA, United States; 6 Department of Pediatrics, Perelman School of Medicine, University of Pennsylvania, Philadelphia, PA, United States

**Keywords:** congenital hyperinsulinism, diabetes, family burden, hypoglycemia, pancreatectomy, quality of life, rare disease, pancreatic surgery

## Abstract

**Introduction:**

Congenital hyperinsulinism (HI) is a rare disorder characterized by severe, recurrent hypoglycemia. Subtotal pancreatectomy remains a treatment option for diffuse HI, but post-surgery quality of life is largely undescribed.

**Methods:**

This mixed-methods study utilized quantitative data from the HI Global Registry, including individuals with diffuse HI and ≥75% pancreatectomy (n = 34). Of these, 13 (38%) completed qualitative interviews to capture the patient and caregiver perspectives. This is the first study to investigate long-term clinical and lived impacts of pancreatectomy for diffuse HI as reported by affected families.

**Results:**

Most participants underwent subtotal pancreatectomy before two months of age (24, 71%). Only three (9%) had normal glucose status at discharge. At follow-up by median (range) age 9 (1.5-32) years, diabetes was reported in 15 (44%), pancreatic insufficiency in 14 (41%), and 24% reported ongoing medication use for hypoglycemia. Continuous glucose monitoring data demonstrated suboptimal time in range, regardless of diabetes status. Interviews revealed variability in surgical decision-making based on preoperative medical management, genetics, and imaging of the pancreas. Hospitalization and post-discharge periods were described as stressful and challenging to mental health for caregivers. At follow-up, children reported less perceived burden than caregivers. Of the six parents interviewed whose children have transitioned to diabetes, four stated that diabetes was easier to manage than HI, although hypoglycemia could still be frequent.

**Discussion:**

Subtotal pancreatectomy for diffuse HI may be necessary when medical therapy is not sufficient to prevent severe hypoglycemia. However, surgery was not curative and was associated with life-long consequences and management.

## 1 Introduction

Congenital hyperinsulinism (HI) is the leading cause of persistent hypoglycemia in infants and children, affecting approximately 1 in 28,000 births (1, 2). HI results from insulin hypersecretion by pancreatic β-cells and may cause seizures and neurodevelopmental disability if untreated (3–8). Therefore, early diagnosis, along with best practices to manage hypoglycemia, are imperative to prevent long-term neurologic complications.

The two major histological HI subtypes, focal and diffuse, have a largely indistinguishable clinical presentation (9), whereas less common atypical HI variants have a different histology and clinical profile (10–12). Focal HI has a high cure rate following surgical removal of the lesion (8, 10, 13). In contrast, diffuse HI, most frequently caused by inheritance of biallelic K_ATP_ channel gene variants, often requires complex medical regimens and, in some cases, pancreatectomy (8, 9, 11, 14, 15). Currently available medical therapies vary in effectiveness, and side effects may restrict use (15–17). When dietary and medical therapies are insufficient to prevent hypoglycemia in diffuse HI, pancreatectomy may offer a better strategy to decrease the frequency and severity of hypoglycemia capable of inducing brain damage (14, 15). Independent to the treatment approach, daily management of HI places physical, mental, and financial burdens on people with HI and their caregivers (16–18).

Because of the complexity of HI, optimal evaluation and treatment require an experienced multi-disciplinary team, including geneticists, endocrinologists, radiologists, pathologists, and surgeons (10, 19). The Congenital Hyperinsulinism International (CHI) Centers of Excellence (COE) program recognizes institutions that have an established multidisciplinary team with the highest commitment to research and best practices. For example, all COEs have access to specialized imaging technology, such as ^18^fluoro-dopa (F-DOPA) positron-emission tomography (PET)-computed tomography (CT) for pre-operative localization of focal lesions and access to intraoperative examination of the histology through frozen biopsies to guide the surgical approach (9, 20–23).

While the outcomes of pancreatectomy for diffuse HI are well described from the clinicians’ perspective (6, 10, 11, 24–26), the patient and family perspective is underrepresented in the literature. Patient-reported research provides insights from lived experience that complement clinical measures to aid in identifying opportunities for better care and informing the measurement of meaningful clinical outcomes. We aimed to describe surgery data for participants in the HI Global Registry (HIGR) with diffuse HI, including the decision-making process regarding surgery; to quantify post-pancreatectomy health outcomes; and to report the psychosocial and quality-of-life impacts of pancreatectomy as described by people with HI and their caregivers.

## 2 Methods

**Figure 1:**
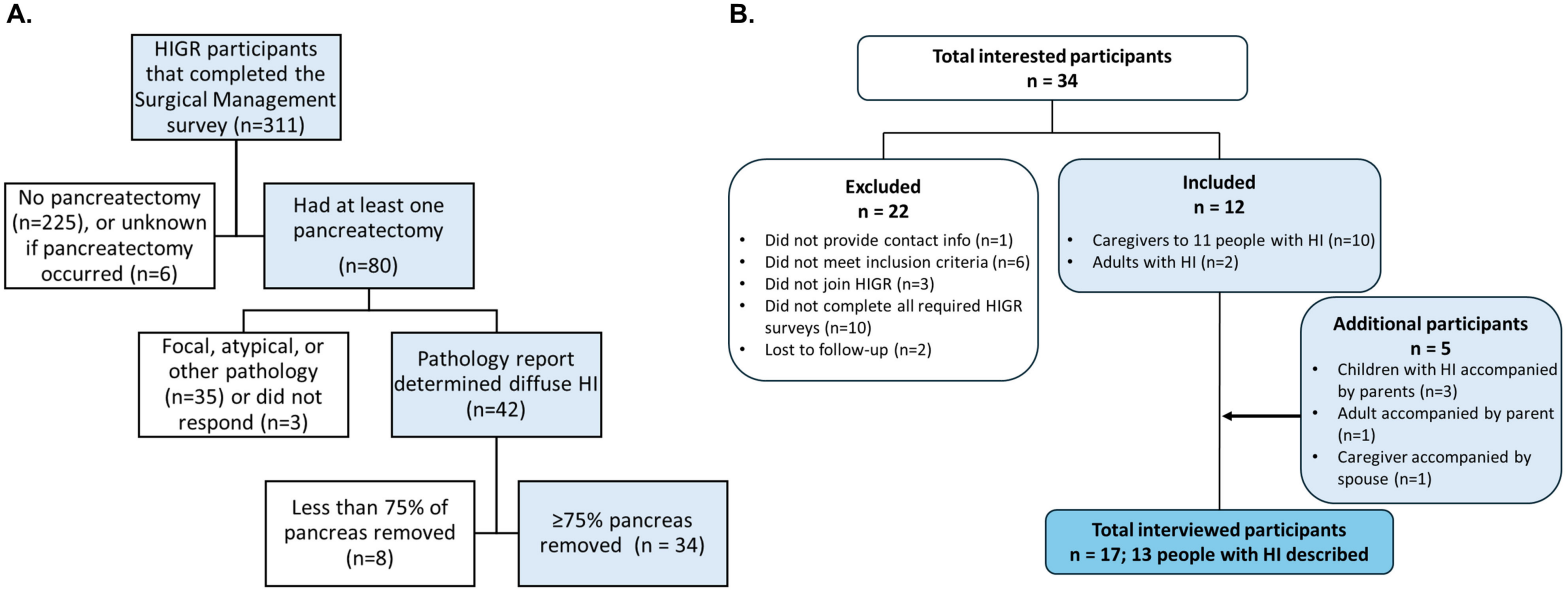
Study inclusion criteria. The flowcharts shown depict the inclusion criteria for the HIGR study **(A)** and the qualitative interviews **(B)**.

In this mixed-methods study, we combined quantitative data derived from HIGR supplemented by qualitative interviews in a subgroup of participants. HIGR is a global web-based, patient-powered registry, developed and maintained by CHI. HIGR provides insight through cross-sectional and longitudinal surveys collecting data about living with HI. Information about the data in HIGR and development of the surveys has been described in previous publications (16, 18, 27). Data were captured from the *Surgical Management* survey completed by September 2025 (n=311). Individuals were eligible to participate in the study if they or their child had undergone ≥75% pancreatectomy and if they indicated that the pathology report determined diffuse HI. Of surgically treated individuals with diffuse HI in the registry, 34 (81%) were included in the current study (**Figure 1A**).

For the interviews, participants were recruited through CHI’s website, social media, and email list. To participate, adults were required to sign an electronic consent form and be willing to complete a one-time 60-minute web-based interview in English. Children over age 12 were invited to participate in a 30-minute interview if they signed an assent form and a parent was present. Among 34 interested people, 10 caregivers and two adults with HI who completed the intake form were included (**Figure 1B**). Three of the caregivers participated with their child, one adult with HI was accompanied by their parent, and one caregiver was accompanied by their spouse, resulting in five additional interview participants. Some caregivers discussed multiple children with HI. In total, 17 participants were interviewed, and 13 people with HI were described. Interview participants lived in Canada, Denmark, Sweden, the United Kingdom, and the United States (**Table 1**). Participant characteristics are summarized to prevent identifiability. Data saturation was reached with the participants interviewed.

**Table 1:** Demographic and clinical characteristics of participants with HI in HIGR and in the qualitative interviews.

Demographics and genetics	People with HI reported through HIGR n (%)	People with HI described in interviews^b^ n (%)
N	34	13
Sex		
Male	16 (47%)	6 (46%)
Female	18 (53%)	7 (54%)
Current age, n (%)^a^		
< 1 year	1 (3%)	0 (0%)
1–3 years	5 (15%)	2 (15%)
4–6 years	6 (18%)	2 (15%)
7–9 years	5 (15%)	3 (23%)
10–13 years	6 (18%)	2 (15%)
14–17 years	5 (15%)	2 (15%)
≥ 18 years	6 (18%)	2 (15%)
Continent of residence, n (%)		
Africa	1 (3%)	0 (0%)
Europe	5 (15%)	3 (23%)
North America	26 (76%)	10 (77%)
South America	2 (6%)	0 (0%)
Race, n (%)		
Asian	1 (3%)	1 (8%)
Black or African American	1 (3%)	1 (8%)
White	23 (68%)	8 (62%)
More than one race	1 (3%)	1 (8%)
Other	2 (6%)	1 (8%)
Unknown or not reported	6 (18%)	1 (8%)
Genetic testing results, n (%)		
K_ATP_ channel variant(s)	24 (71%)	9 (69%)
Positive for variant(s) in HI gene, but specific gene was not reported	5 (15%)	3 (23%)
No genetic testing conducted	4 (12%)	0 (0%)
Unknown if genetic testing was conducted or results of testing unknown	1 (3%)	1 (8%)

^a^ Current age refers to the age at most recent survey submission or at the time of interview.

^b^ All individuals who participated in the interviews also completed HIGR and are reported within that group.

There were 27 HIGR participants that responded to whether they identify with a community known to have a higher incidence of HI. Five (19%) indicated belonging to the Ashkenazi Jewish community. Of 33 HIGR participants who responded to whether they have a syndrome known to occur with HI, one reported having Beckwith-Wiedemann Syndrome.

Interview scripts utilized open-ended questions, with probing follow-up questions for additional thematic exploration. Qualitative data analysis utilized a modified grounded-theory approach, including immersive reading, open coding, consensus coding, thematic identification, peer debriefing, and interpretative analysis (28, 29). Two authors completed coding using Delve, a qualitative data analysis software program. The coding, peer debriefing, and contextualization process for the final paper were consistent with the methodology described previously (16). Peer debriefing was conducted by three individuals, including those who were and were not familiar with HI and pancreatectomy, to ensure clarity of interpretation of the data and reproducibility.

Interviews were conducted between April and September 2025. Caregivers and adults who participated in interviews received a $125 gift card, and parents of the children who participated were given an additional $75 for their time. Each participant’s current age is reported as that at their most recent survey completion or at the time of interview. Interview participants were assigned an identifier to ensure anonymity. Quotes were edited for clarity without changing their meaning and were attributed to the caregiver (CG) or the person with HI (HI) with a unique code.

HIGR participants included in the study had the opportunity to share data from continuous glucose monitoring (CGM; Dexcom^©^, any generation). Primary outcome metrics included mean glucose (mg/dL), glycemic variability (coefficient of variation), time in range (TIR; 70–180 mg/dL), time below range (TBR; <70 mg/dL), and time above range (TAR; >180 mg/dL). The percent of CGM wear time was analyzed with two methodologies. First, wear time was calculated as percent of time across days of data availability, where full days of data gaps were excluded; additionally, total wear time was calculated as percent of time from the first day of reported CGM use to the last day of reported CGM use or September 12, 2025. Data were summarized and visualized using Posit Software, PBC RStudio (version 2025.05.1 + 513). Given the exploratory and descriptive nature of this study and the limited sample size, analyses across the study were intended to characterize participant experiences and/or glycemic data rather than to perform formal statistical analyses.

The study was approved by the North Star Review Board (NB500283).

## 3 Results

### 3.1 Participants

Of the 34 included participants who completed the *Surgical Management* survey in HIGR, 50% were over age 10, 76% were from North America (of whom 96% were from the United States), and 68% were White (**Table 1**). All participants who had genetic testing performed and reported on the results indicated variants in K_ATP_ channel genes (**Table 1**). Most participants had their first pancreatectomy before 2 months of age (71%), and 85% had more than 95% of their pancreas removed in total (**Table 2**). The median (range) age at follow-up in HIGR was 9.5 (0–35) years, and in the interview part, 9 (1.5-32) years. In the interviews, one individual reported multiple surgeries, and seven were diagnosed with post-pancreatectomy diabetes.

**Table 2:** Surgery and discharge data as reported in HIGR.

Surgery and discharge data	n (%)
Were any of the following radiology/imaging tests performed on the participant to diagnose or confirm HI and/or the subtype?^a^	
None	14 (41%)
^18^F-DOPA PET Scan	14 (41%)
Ultrasound	2 (6%)
CT or MRI scan	2 (6%)
Imaging with other tracers	2 (6%)
Unknown	5 (15%)
Age at first pancreatectomy surgery	
< 2 months	24 (71%)
2–3 months	4 (12%)
4–11 months	3 (9%)
≥1 year	3 (9%)
Total amount of pancreas removed during surgery	
75-94%	5 (15%)
95% or more	29 (85%)
Did the participant experience any complication(s) during the pancreatectomy?	
No	25 (74%)
Yes^a^	9 (26%)
Bowel damage	*2 (6%)*
Bile leak	*2 (6%)*
Wound infection	*1 (3%)*
Hemorrhage	*1 (3%)*
Fistula formation	*1 (3%)*
Anesthesia problems	*1 (3%)*
Other^b^	*3 (9%)*
Did the participant require more than one pancreatectomy?	
No additional pancreatectomy was necessary	25 (74%)
An additional pancreatectomy was performed before discharge from the initial surgery	5 (15%)
An additional pancreatectomy was performed after discharge from the initial surgery	4 (12%)
Outcome at the time of discharge following the final pancreatectomy	
Required HI medication AND frequent, continuous or special feeding for low blood glucose	14 (41%)
Required frequent, continuous or special feeding for low blood glucose, no HI medication	11 (32%)
High blood glucose, required insulin	6 (18%)
Normal blood glucose, no medication or special feeding required	3 (9%)
Amount of time between discharge from final pancreatectomy and change in HI management	
No change necessary	2 (6%)
Less than 1 month	9 (26%)
1–3 months	8 (24%)
4–6 months	6 (18%)
7–9 months	1 (3%)
10–12 months	3 (9%)
Greater than 1 year	2 (6%)
Unknown	3 (9%)

^a^ Multiple options could be selected.

^b^ Included wound dehiscence, cholodocoduodenostomy, and an unspecified complication.

### 3.2 Disease at onset, decision to have surgery, and preoperative imaging

In the interviews, parents described their babies’ symptoms that led to a diagnosis of HI. Early indicators included seizures, refusal to eat, lack of crying, purple discoloration of the skin, lethargy, cold skin, and shakiness. Interview participants described the initial treatments to stabilize blood glucose, including dextrose administered through an intravenous (IV) or a peripherally inserted central catheter (PICC) line, glucagon, steroids, and continuous feeds.

**Figure 2:**
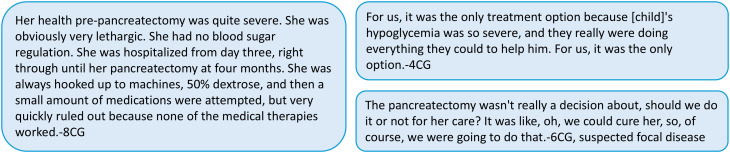
Pre-surgical health status and perception of surgery. Additional quotes from the interviews related to pre-surgical health status and the perception of surgery are presented. In the participant identifier, CG refers to the caregiver of someone with HI.

Prior to pancreatectomy, trials of diazoxide were described by all interviewed caregivers. Only two tried off-label octreotide; one parent declined, and the others were not offered octreotide. The medications were either ineffective at managing hypoglycemia or caused severe side effects, including elevated heart rate (on diazoxide). In caregiver interviews, families described repeated attempts at medical management prior to surgery and the associated burden of side effects and persistent hypoglycemia (**Figure 2**).

The first four months of his life, we were continuously in hospital … They tried everything. They tried diazoxide, and then they started to use octreotide … Meanwhile, he’s on continuous feed, continuous glucose drip, and still, that’s not enough. Still, the sugars are too low.-13CG.

**Figure 3:**
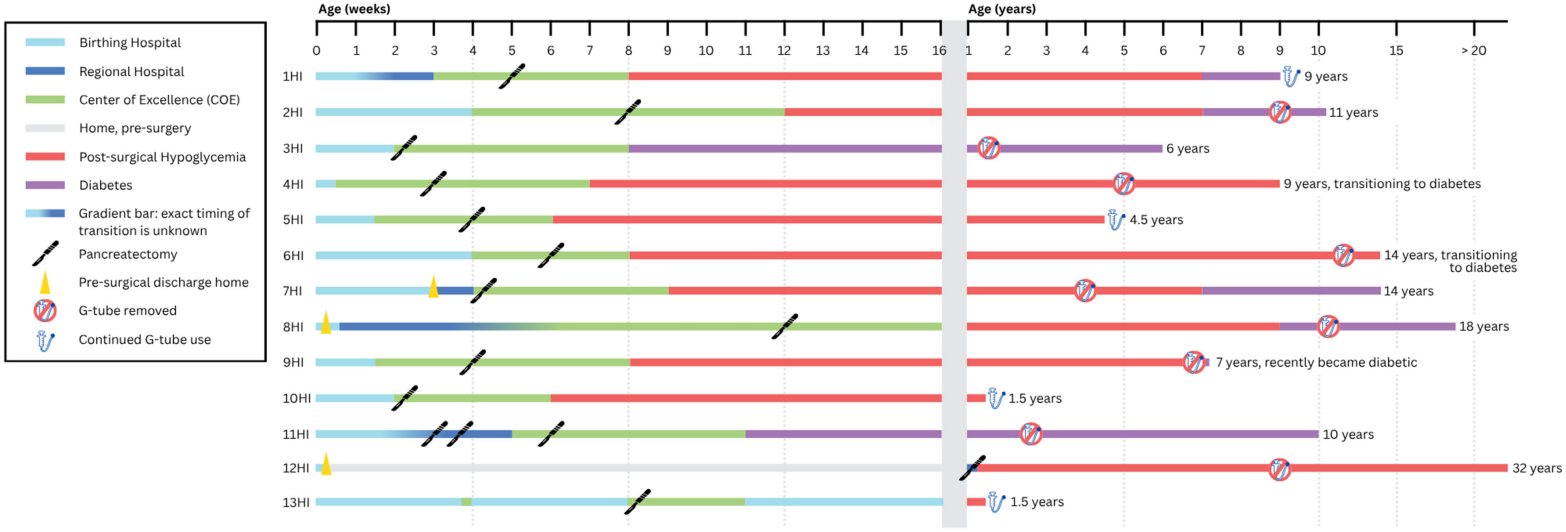
Care journeys of people with HI described in the interviews. Each row represents an individual described in the interviews with corresponding time spent (weeks or years) in each phase of the care journey, followed by the age at the time of interview and relevant clinical information, as appropriate. The legend provides the colors and symbols used to describe the major clinical events, placed at the time of the event, for each individual.

Six interview participants specifically noted that the birthing hospital “exhausted all resources” or lacked the HI expertise to stabilize the child’s blood glucose. Five individuals were transferred to larger regional children’s hospitals, one individual was born in the regional children’s hospital and briefly visited a COE where they later returned for surgery, and seven were transferred directly to a COE (**Figure 3**). In the end, all but one individual was eventually transferred to a COE.

Caregivers were asked if pancreatectomy was presented as the only treatment option or if continuing medical management was offered as an alternative. Five said that pancreatectomy was described as the only option based on the inability to achieve glycemic stability. The others described a decision to have a pancreatectomy as inevitable after other options had been exhausted, suggesting medical management was trialed.

They were trying everything else first –feeds, diazoxide, all sorts of stuff—which ended up making things worse rather than better. Then they said, “Okay, now it’s time to consider surgery,” but it was definitely not presented as an original first-best option.-11CG.

Some caregivers decided to pursue surgery for their child based on what they were told about their child’s type of HI following preoperative imaging, which afforded hope that the surgery would be curative. In the *Surgical Management* survey in HIGR, 59% of participants reported undergoing radiology or imaging tests to diagnose their HI subtype, including 41% who had an ^18^F-DOPA PET scan, all of which were completed at a COE (**Table 2**). Of the 14 HIGR participants who had the ^18^F-DOPA PET scan, five reported that the images suggested focal HI, but only one of the five reported a single, paternally inherited recessive K_ATP_ channel variant; however, surgical pathology indicated diffuse disease requiring 98% pancreatectomy for this individual.

Four participants who were interviewed stated that they were told their child could have focal disease, based on imaging results. Further, two individuals described a lack of clarity regarding the scans, including disagreement between members of the medical team that remained unresolved until after surgery (**Figure 2**).

The doctor (endocrinologist) looked at his (^18^F-DOPA) PET scan and said, “This one is so easy, even a monkey could read this. This is a focal lesion.” We’re like, “This is the best news.” The radiologist had looked at it, and the radiologist thought, “That looks diffuse to me.” Obviously, they got in, and it ended up being diffuse.-7CG.

### 3.3 The pancreatectomy surgery and in-hospital recovery

**Figure 4:**
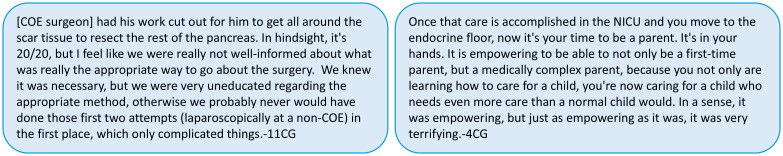
The pancreatectomy surgery and in-hospital recovery. Additional quotes from the interviews related to pancreatectomy surgery and in-hospital recovery are presented. In the participant identifier, CG refers to the caregiver of someone with HI. COE, Center of Excellence; NICU, neonatal intensive care unit.

In HIGR, 71% of children were younger than 2 months at the time of their first surgery (**Table 2**). Those who were interviewed recalled stress related to the day of surgery, and two admitted concerns that their child would die. While eight children had both parents present on surgery day, some parents could not be present due to work or childcare needs, which put added stress on the family. Participants used words including “terrified,” “worried,” “nervous,” “a wreck,” “confused,” “like a shell,” “alone,” “anxious,” and “helpless”; two individuals cried when recounting the surgery day (**Figure 4**).

They’re taking this big portion out of my child. It was heartbreaking, and he’s just so little and newborn. It’s heartbreaking to hear that as a new mother. It was overwhelming -1CG.

In HIGR, 41% of participants reported having a gastrostomy for G-tube placement performed during their pancreatectomy. While most individuals described in the interviews had the G-tube placed during the pancreatectomy, four had it placed after the surgery due to miscommunication about the preference to have it inserted or based on optimism that it would be unnecessary. In the end, all participants described in the interview study used a G-tube following surgery, and nine had it removed by the time of interview (**Figure 3**).

Surgical complications were reported by 26% of HIGR participants, most commonly involving bowel damage or bile leak (**Table 2**). Four individuals from the interviews experienced surgical complications. Two had complications with the surgical wound, and two experienced bile duct complications. In one case, this was identified during the surgery, necessitating a choledochoduodenostomy. In the other case, the individual developed pain nine months post-pancreatectomy. Still, the cause, a biliary system leak which necessitated a cholecystoduodenostomy, was not identified for another year.

Nine (26%) participants reported multiple pancreatectomies in the HIGR survey (**Table 2**), including two who had three resections. In the interviews, two individuals discussed laparoscopic surgical approaches. One was converted to an open laparotomy; the other individual underwent two unsuccessful initial laparoscopic surgeries followed by a later laparotomy performed at a different institution (**Figure 4**).

Those who were interviewed referred to the recovery period before discharge as particularly challenging, including five who recounted the difficulty of seeing their infant in distress.

You could tell that she was in pain or hurting, and there was nothing that they could do for her. That was really hard.-6CG.

In addition to wound care and pain mitigation, the postoperative recovery period focused heavily on glycemic control and feeding to establish a management plan ensuring safe post-discharge glucose levels. While glycemic control was highly variable during this period, all those interviewed noted an improvement after the final surgery compared to before. Feeding was frequently described as a necessary step in preparing for discharge, often supported by the use of a G-tube for continuous or bolus feeds. However, feeding challenges were common; several caregivers described heightened stress due to pressure from hospital staff, which subsided when the environment changed.

It was a huge difference because before the surgery, he wasn’t stable even though he had continuous feeds … It took about, all in all, two months for us to be able to come home, for him to be stable enough to not need any continuous sugar, any continuous food, and to remain stable by feeding him every three hours throughout the day and then continuous feeds throughout the night.-13CG.

I remember the doctor overseeing her care at the time was very aggressive with pushing the feeds. I remember having to have a conversation with him and tell him, “Look, you’re making things worse because you’re pushing it too fast.” As soon as he did listen to me and we slowed down things, then it only took a day or two more before she was able to tolerate feeds and leave the NICU.-2CG.

On average, families interviewed spent a total of two months in the hospital (**Figure 3**), which was described as exhausting. Some parents noted mental health challenges, including depression. During recovery, some individuals appreciated being transferred from the NICU/PICU post-surgery to specialized endocrine floors with knowledge of HI to help prepare for the reality of caring for their child at home (**Figure 4**).

### 3.4 Transition to home and follow-up care

Once families were discharged home, they faced the adjustment to home life with an infant with a complex medical condition, which interviewees described as “terrifying,” “high-stress,” “brutal,” “chaos,” and “a full-time job” due to the need for hypervigilance.

You’re just so afraid that you’re going to do something wrong and ruin the rest of his life … There was just a learning curve that was super steep—you felt like at any moment you’re going to do something that was going to be life-altering.-11CG.

**Figure 5:**
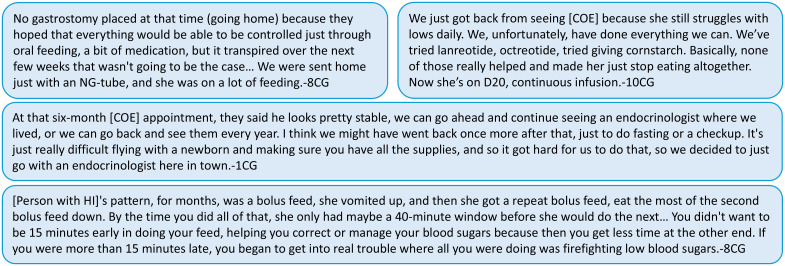
Transition to home and site of medical management. Additional quotes from the interviews related to the transition to home and site of medical management are presented. In the participant identifier, CG refers to the caregiver of someone with HI. COE, Center of Excellence; D20, 20% Dextrose; NG, nasogastric.

Frequent adjustments to the treatment regimen were common due to fluctuating glucose management needs after the transition home (**Figure 5**). At discharge, most individuals received a combination of medication and feeding regimens, with only 27% of HIGR participants having reported post-pancreatectomy resolution of hypoglycemia (**Table 2**). However, a change in medical management was reported by 50% of HIGR participants within three months post-pancreatectomy. In interviews, caregivers described frequent adjustments and uncertainty during the transition to home care.

When we were sent home, we were on 24/7 dextrose feeds. Because [his glycemic level] was all over the place, that was our safest route. At the time, they didn’t really introduce too many medications to us. I feel like it was up in the air with the route they wanted to take.-4CG.

I think it took about a month or so of just life being different at home. I feel like whenever a kid’s at the hospital, they behave differently.-2CG.

Many of those interviewed continued follow-up care with the endocrine team, for at least some period, at the hospital where the pancreatectomy was performed. Three individuals remained under the care of their COE (**Figure 5**). In some cases, the local providers and families themselves maintained active communication with COEs for additional support. A few families noted being their local provider’s only HI patient.

### 3.5 Management of ongoing hypoglycemia

Two individuals in the interview study were discharged with diabetes after pancreatectomy, but everyone else experienced a period of hypoglycemia (**Figure 3**). Three managed hypoglycemia with lanreotide or octreotide, and two were enrolled in a clinical trial for an investigational medication. Interviewees noted trade-offs with each medication. One family celebrated stopping four-time daily injections of octreotide but called the monthly lanreotide shots “traumatic”; other participants reported side effects impacting other organs. All individuals on medication also relied on dextrose via G-tube or feeding as part of their overall glucose management (**Figure 5**).

She was put on octreotide around 2 years old, and at the same time, a G-tube was put in. We first started with octreotide shots, and that didn’t work. Eventually, she got onto the pump, and that helped a lot. I think we were able to do feeding every three hours.-12CG.

Those who took HI medications expressed challenges with inconsistent dosing, rapid glucose shifts, and the variability of blood glucose levels, and noted that none of the currently available treatments adequately controlled their child’s hypoglycemia. At the time of survey, 24% of HIGR participants reported taking HI medications—all somatostatin analogs (**Table 3**). Of the eight participants taking an HI medication, all discussed dysglycemia, including three who reported hypoglycemia at least once per day and six who reported hyperglycemia at least once per week.

**Table 3:** Medication use and dysglycemia post-pancreatectomy as reported in HIGR.

Medication use and dysglycemia	n (%)					
Is the participant currently taking an HI medication?						
Yes^a^	8 (24%)					
No	23 (68%)					
Not reported	3 (9%)					
Did the participant report having a diabetes diagnosis?						
Yes	15 (44%)					
No	12 (35%)					
Unknown or not reported	7 (21%)					
At what age was the participant diagnosed with diabetes? (n=14)^b^						
Immediately after surgery	1					
<7 months	2					
7–12 months	2					
1–3 years	0					
4–9 years	5					
10–15 years	3					
>15 years	1					
How is the participant’s diabetes currently managed?^c^						
Insulin pump	9 (60%)					
Diet	6 (40%)					
Multiple daily injections	6 (40%)					
Metformin	0 (0%)					
Frequency of dysglycemia events by group						
	Taking an HI medication and not diagnosed with diabetes (n=8)		Not taking HI medication and not diagnosed with diabetes (n=8)		Diagnosed with diabetes (n=15)	
Frequency of dysglycemia	Hypo-glycemia	Hyper-glycemia	Hypo-glycemia	Hyper-glycemia	Hypo-glycemia	Hyper-glycemia
At least once per day	3	1	2	3	4	13
At least once per week	4	5	2	1	9	1
At least once per month	0	1	3	2	1	0
Infrequently	1	1	1	2	1	0
Never	0	0	0	0	0	0
Unknown	0	0	0	0	0	1

Hypoglycemia: Blood glucose level below 70 mg/dL (3.9 mmol/L, 0.7 g/L).

Hyperglycemia: Blood glucose level above 180 mg/dL (10 mmol/L, 1.8 g/L).

^a^ Participants reported taking octreotide (n=2), octreotide and steroids (n=1), long-acting octreotide (n=2), and lanreotide (n=3).

^b^ One participant who has diabetes is not reported in this table due to an unclear timeline of the diagnosis of diabetes compared to time of surgery.

^c^ Participants could select more than one response; therefore, the total will not equal 100%.

His blood sugars were anywhere between 400 (mg/dL) and 40 (mg/dL) in a given day on medication.-9CG.

She was exclusively using lanreotide and food. It wasn’t perfect. The lanreotide shots would increase her blood sugar for a few days and then didn’t always sustain it as long as we wanted.-6CG.

### 3.6 Transition to post-pancreatectomy diabetes

For those who did not immediately transition to diabetes, there was a period described in the interviews as the “honeymoon phase” or “holding pattern,” where blood glucose was managed without medication; however, three interviewees expressed uncertainty when this period would occur and if it would last for months or years. During this period, some caregivers reported closely monitoring HbA1C and used diet as their management strategy. However, of the 17 HIGR participants who had not been diagnosed with diabetes, only 59% reported having HbA1C testing as part of their regular check-up. In the period with no HI medication but prior to diabetes diagnosis (n=8), all participants reported dysglycemia (**Table 3**). Two reported experiencing hypoglycemia daily, and three reported daily hyperglycemia. Importantly, all HIGR participants reported hypoglycemia and hyperglycemia to some extent, regardless of medication or diabetes status.

Right now, [person with HI] is pretty controlled with diet. We get the occasional low … I’ll say maybe once a week. Our biggest medical issue right now are the high blood sugars. We are monitoring his A1C, which has been pretty elevated for the last nine months.-4CG.

The transition to diabetes was indicated based on blood glucose levels or HbA1C. In HIGR, 44% of participants reported a diabetes diagnosis (**Table 3**); the majority were diagnosed between ages 4 and 9. In the interviews, the median (range) time from surgery to diabetes was 7 years (5 weeks to 9 years). All HIGR participants who reported a diabetes diagnosis indicated dysglycemia even after treatment for diabetes began, including four who experienced hypoglycemia and 13 who experienced hyperglycemia at least once per day (**Table 3**).

I think it was [age] 7, he started having high blood glucoses along with his lows, but his lows are more predictable. Maybe he’d have like three, four lows a day, but then he was also getting highs, really high highs.-1CG.

Individuals emphasized in the interviews that the greatest diabetes management challenge was ongoing hypoglycemia and blood glucose variations. Participants described a combination of strategies to manage their diabetes; importantly, none reported using diet alone (**Table 3**). Several caregivers noted that the clinical knowledge and experience with diabetes was more widespread compared with HI, making diabetes care relatively easier.

We’re getting used to the insulin and figuring that part out. [COE nurse] always said it was easier than the HI part, and I think she’s right.-9CG.

CGM data shared by eight HIGR participants with diabetes showed TIR below the benchmark of 70% (30) in the majority of participants, driven by a high TAR (**Supplementary Table 1**). Two participants reached the TIR threshold when only their last month of data was considered, but none met this criterion based on their historical data. However, most participants had TBR levels below the clinical benchmark of 4%.

The insulin does not always work because the diabetes that I have isn’t as predictable as regular diabetes. Sometimes we will do the correct amount [of insulin], but it will still just be high during the night. That can sometimes affect me. Then during the day, I would say being active can make my sugar go low.-2HI.

### 3.7 Feeding

Feeding/eating played a crucial role in glycemic control for all interviewed families and was a prominent theme throughout the care journey. Both the timing and composition of meals were key factors for all participants, though timing of feeding was often described as more consequential when managing HI. Some interviewees said they maintained a consistent and often limited diet to reduce glucose level variability. However, others noted that glycemic control remained unpredictable even with consistent dietary intake.

You could do the same thing from day one to day two, and she could eat exactly the same things, and her blood sugars would be totally different … There was no continuity to this disease and how it reacted in her body.-6CG.

Feeding/eating issues were reported by 59% of HIGR participants and were similarly described as a major challenge for all but one caregiver interviewed (**Table 4**). Parents stated that these difficulties were most prominent in the newborn period, when they were still managing hypoglycemia and feeding was critical to achieving euglycemia. Vomiting was often linked to feed volume or “force-feeding,” while food refusal and slow eating complicated mealtimes, increasing reliance on the G-tube. Additionally, some caregivers reported that their children struggled to gain weight. One adult with HI recalled anxiety about the physical process of eating.

**Table 4:** Feeding routes and feeding issues as reported in HIGR.

Feeding routes and feeding isuues	n (%)
Has the participant utilized tube feeding since HI was suspected?^a^	
Yes	29 (85%)
No	2 (6%)
Not reported	3 (9%)
Has the participant experienced any feeding issues on a regular basis?	
Yes	20 (59%)
No	9 (26%)
Not reported	5 (15%)
Feeding issue reported (n=20)	
Poor appetite	14 (70%)
Slow eating	14 (70%)
Refusing to eat	11 (55%)
Problems with texture	9 (45%)
Gagging	7 (35%)
Coughing	6 (30%)
Vomiting	6 (30%)
Reflux	6 (30%)
Uncoordinated oral skills	6 (30%)
Overeating	3 (15%)

^a^ Tube feeding includes nasogastric (NG)/orogastric (OG), G-tube or button, J-tube, or PEG.

^b^ Participants could select more than one response; therefore, the total will not equal 100%.

Even the action of chewing, swallowing, I had to learn all of that. It wasn’t instinctive. At first, it created a lot of anxiety to swallow and not get food stuck because it was hard … After I got the help, it got better at 9, 10 years old.-12HI.

To manage feeding difficulties, families discussed strategies such as allowing TV time, using puppets, celebrating every bite, and telling jokes. While these techniques worked for some, others recalled stress that disrupted family dynamics.

It’s something that is hard to remember all those years fighting for her to eat, and the stress around that. It didn’t help our relationship. The family relationship, the family dynamics were awful around that period because of the feeding issues.-12CG.

To address feeding challenges, eight interviewees enrolled their child in feeding therapy or worked with a psychiatrist, occupational therapist, or in-home nurse specializing in feeding. Five attended outpatient clinics, while three completed extended inpatient programs. Among the 20 HIGR participants who reported feeding issues, 75% received feeding therapy. Of those, 50% noted that their issues had not resolved. However, details of the type and duration of feeding therapy were unknown.

### 3.8 Exocrine pancreatic insufficiency

In HIGR, 41% of participants reported a diagnosis of EPI, including 38% who were taking pancreatic enzymes (**Table 5**). Of those who took pancreatic enzymes (n=13), 53% began within one year of surgery, while others started taking pancreatic enzymes later in life. Some interviewees noted that their endocrinologist automatically prescribed the medication because of undergoing a subtotal pancreatectomy, while others noted ongoing monitoring to identify the need. Interviewees mentioned the difficulty of taking the capsules at every meal, especially for small children, who needed them opened and sprinkled onto their food.

**Table 5:** Exocrine pancreatic insufficiency as reported in HIGR.

Exocrine pancreatic insufficiency	n (%)
Has the participant been tested for exocrine pancreatic insufficiency?	
Yes	18 (53%)
No	13 (38%)
Not reported	3 (9%)
Has the participant been diagnosed with exocrine pancreatic insufficiency?	
Yes	14 (41%)
No	17 (50%)
Unknown	3 (9%)
Does the participant take pancreatic enzymes?	
Yes	13 (38%)
No	18 (53%)
Unknown	3 (9%)
Time between pancreatectomy surgery and starting pancreatic enzymes (n=13)	
Less than 3 months	5 (38%)
4–12 months	2 (15%)
1–3 years	3 (23%)
4–9 years	1 (8%)
10+ years	2 (15%)

### 3.9 Dysglycemia in daily life

While two interviewees reported predictable glycemic control patterns for themselves or for their child, most indicated unstable blood glucose levels. Factors causing variation included extreme weather, hormones (including menstruation), growth spurts, and illnesses.

She’ll have fluke days where she’s just borderline low, maybe hovers around 70 [mg/dL]. I’ve determined those are maybe growth spurt type days or just a total different surge of hormones that day, and so she’s a little lower.-5CG.

**Figure 6:**
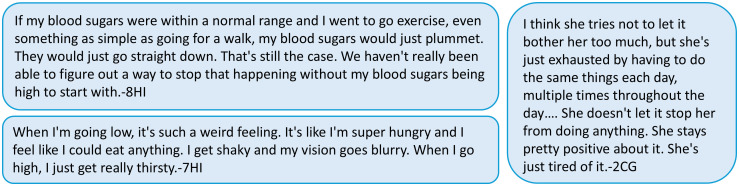
Dysglycemia symptoms and impact on activity. Additional quotes from the interviews related to dysglycemia symptoms and impact on activity are presented. In the participant identifier, CG and HI refer to caregiver or person with HI, respectively.

The most cited aspect of daily life impacting blood glucose levels was exercise. Regardless of current medical status, many people interviewed described exercise or activities causing blood glucose to “plummet.” To avoid the rapid drop, some restricted exercise to certain times of day, started exercise with high blood glucose levels, or stopped activity for a bolus or snack (**Figure 6**).

I remember being frustrated because we wanted to go for a walk. It was a nice day. Let’s go into the woods. It wasn’t 15 minutes into the walk that she was into hypoglycemia. We have to stop, have to feed her.-12CG.

I would have to take time off work to attend every [sports] game because I knew they wouldn’t be watching it closely enough … There were times I would have to literally call her off the court to have sugar [for hypoglycemia], which is unheard of in her daily life [where] we’re just begging for her blood sugar to drop down, and it won’t. With activity, it does. It does too quickly, and it becomes dangerous.-2CG.

A few caregivers interviewed could not easily identify signs of hypoglycemia when the child was young, but over time, noticed their child wanting to lie down, feeling groggier, experiencing fatigue, or exhibiting more challenging behaviors during hypoglycemic episodes. However, not all individuals experienced hypoglycemia symptoms. Individuals who could identify symptoms used phrases like “lightheaded,” “energy level goes down,” “dissociated,” and “more emotional,” and described headaches, tingling, or blurry vision during hypoglycemia. Individuals had fewer comments regarding hyperglycemia, but one reported headaches and another extreme thirst (**Figure 6**).

He’s been very asymptomatic, where we wouldn’t know, and we just see it from the CGM. Then, as he got a little bit older, like 5, 6, 7, we could see him getting worse behavior, angry, more acting out.-9CG.

It’s a lot more cognitive than is talked about … if I went really high yesterday, I could still feel those effects today. You can very easily get more emotional or headaches, irritated, tired, dizzy … sometimes it’s harder to focus.-2HI.

I would say it would be almost impossible for us to monitor him just taking his [glucometer] measurements … We do use the Dexcom because in just half an hour, he can go from a really good value of 6 [mmol/L] or 7 [mmol/L] up until 3.8 [mmol/L] suddenly.-13CG.

Some of CGM-related challenges included skin irritation and allergies to the tape, the need for constant calibration, concerns about accuracy in the low ranges, false alarms, and accessing supplies through insurance, especially for people who had not transitioned to diabetes.

The CGMs aren’t really built to detect people who are persistently low. We get a lot of failed sensors with her. Rather than lasting 10 days, they usually only last three or four, and then we have to change them because the sensor just doesn’t know how to process someone who’s low all the time.-10CG.

### 3.10 Quality of life

Across interviews and in HIGR, both individuals with HI and their caregivers described quality of life as influenced by the challenges of medical management, the desire to maintain normalcy, and, for children, evolving independence. In interviews, there was also a stark contrast between caregivers’ sense of disruption and children’s own experiences.

From the way they say it, they don’t even think about their condition much. It’s just part of who they are … We’re just projecting our own assumptions about quality of life to them.-11CG.

Children with HI described their lives as “normal” or stated they “didn’t know any different.” The adolescents began acknowledging the challenges of managing their condition, yet tended to normalize their experiences and maintain a positive outlook. With hindsight, the adults with HI could further articulate how their medical needs truly impacted their childhood.

We knew we wanted to get [person with HI] to have responsibility and autonomy over her own condition … We were supervising from a high level. The easier, the better things would be for her, the more she would get to grips with the normalization of this.-8CG.

From a young age, I was always quite aware of what my blood sugars were doing before you [looking at parent] tested them. I could always prick my own finger myself with the glucometer, and I was always doing that with your help. Then that got even more independent as I got older and started managing that even more myself.-8HI.

Individuals with HI spoke fondly of their pediatric medical teams, sometimes describing them like family. However, experiences transitioning to adult endocrinology care varied. One individual with diabetes reported smooth coordination, while an individual still experiencing hypoglycemia struggled to find clinicians with HI experience. Both caregivers and individuals with HI expressed a desire to better understand how HI might affect future life decisions, such as pregnancy.

I felt really abandoned. We had a lot of specialists. Everyone knew me, my case, and my particularities, and then just nothing. I had three different endocrinologists in my adult life. They’re not HI specialists, so I don’t feel I have any support.-12HI.

Some interviewed caregivers noted their child’s self-consciousness about the tools needed to manage their condition, including the G-tube or the CGM. One parent described her child as “like half machine, half baby, half cyber baby.” Prolonged hospitalizations were viewed as disruptive to early development and the cause of missed developmental milestones. Some caregivers mentioned challenges with foundational exercises, such as “tummy time”. Another caregiver attributed severe allergies to prolonged exposure to a sterile hospital environment. Some parents noted improvements in development over time.

There’s a lot of sadness about the impact of her hypoglycemia on her life. There’s jobs that she lost because they didn’t want to give her flexibility on the time that she could eat and all that. That’s tough on her.-12CG.

Caregivers consistently emphasized the importance of school and teachers. Most described teachers and school nurses as supportive partners and recounted positive experiences when educating school staff and classmates about HI or signs of diabetes. Some families used medical daycares or homeschooling for closer oversight. Most children reported feeling included at school but noted disruptions in class when CGMs alarmed, sometimes resulting in missed social or learning opportunities.

In school, the times we had to play were at noon, after eating. I took almost all the time to eat, and I had to watch my other friends play while I had to finish my meal, so that was really, really hard, and that was over and over and over again each day.-12HI.

In school, if my blood sugars went low, I wasn’t able to do the activities that they would have been doing at the time. Whenever I had my gastrostomy tube, other kids would be pointing that out. They were just like, oh, yes, maybe I am a bit more different than everyone else, but I don’t think it ever stopped me from doing anything.-8HI.

**Figure 7:**
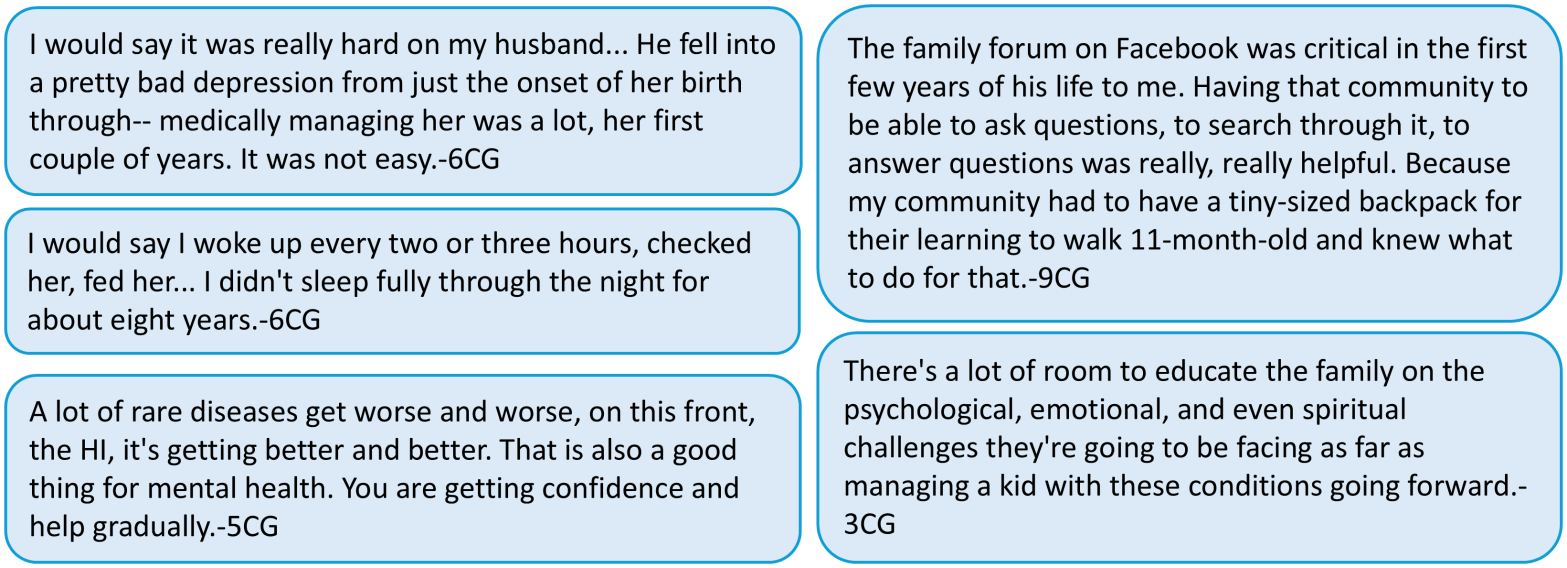
Mental health, quality of life, and support. Additional quotes from the interviews related to mental health and support are presented. In the participant identifier, CG refers to the caregiver of someone with HI.

When caregivers reflected on their own quality of life impacted by their child’s pancreatectomy and ongoing disease management, they discussed the emotional and psychological toll, especially during infancy when hypoglycemia was frequent (**Figure 7**). Cited terms included “traumatizing,” “stressed,” and “nervous breakdown.” In the *Caregiver Quality of Life* HIGR survey (n=25), 24% of caregivers responded “quite a lot” or “very much” when asked if caring for the person with HI affected their physical health, and 32% answered similarly regarding mental health (**Table 6**). These findings were further illustrated in interviews, where caregivers described significant emotional, physical, and financial burdens.

**Table 6:** Caregiver quality of life impacts as reported in HIGR (n=25).

Quality of life impact	n (%) of caregivers						
Impacts on school and work	Do not work outside of home	None	Rarely	Some	Quite a lot	Very frequently	Prefer not to answer
In the last year, how often have you missed school/work due to the participant’s HI?	5 (20%)	3 (12%)	5 (20%)	7 (20%)	3 (12%)	2 (8%)	0 (0%)
	N/A	Poor	Fair	Good	Very good	Excellent	Did not respond
How would you rate the participant’s school in meeting his/her health care needs?	4 (16%)	1 (4%)	3 (12%)	4 (16%)	7 (28%)	5 (20%)	1 (4%)
Impact on physical and mental health		Never	Seldom	Quite often	Very often	Always	Prefer not to answer
Do you feel that your life is ruled by the participant’s HI?		2 (8%)	10 (40%)	5 (20%)	3 (12%)	5 (20%)	0 (0%)
		Not at all	Very little	Somewhat	Quite a lot	Very much	Prefer not to answer
Do you feel your physical health has suffered from caring for the participant with HI?		3 (12%)	6 (24%)	10 (40%)	4 (16%)	2 (8%)	0 (0%)
Do you feel your mental health has suffered from caring for the participant with HI?		2 (8%)	3 (12%)	12 (48%)	4 (16%)	4 (16%)	0 (0%)
Impact on support		Poor	Fair	Good	Very good	Excellent	Prefer not to answer
In general, how would you rate the support you receive from family?		2 (8%)	1 (4%)	7 (28%)	6 (24%)	8 (32%)	1 (4%)
In general, how would you rate the support you receive from healthcare professionals?		0 (0%)	5 (20%)	6 (24%)	9 (36%)	5 (20%)	0 (0%)
	N/A	No impact	Ended relationship	Negative impact	Somewhat stronger relationship	Much stronger relationship	Prefer not to answer
In general, how would you best describe the impact of the participant’s HI on your relationship with your partner?	4 (16%)	2 (8%)	1 (4%)	6 (24%)	5 (20%)	6 (24%)	1 (4%)
Impacts on costs		Very poor	Poor	Fair	Good	Very good	Prefer not to answer
How would you rate your ability to pay for the costs associated with caring for the participant with HI?		0 (0%)	2 (8%)	11 (44%)	7 (28%)	5 (20%)	0 (0%)
		Unable to afford even with financial assistance	Need financial assistance	Difficult	Manageable	Has not affected	Prefer not to answer
How has caring for the participant with HI affected your ability to pay for household essential expenses?		0 (0%)	2 (8%)	11 (44%)	7 (28%)	5 (20%)	0 (0%)

N/A = Not applicable.

Sleep disruption also posed a major challenge. Nighttime CGM alarms and frequent feeding interventions contributed to chronic fatigue even once children transitioned to diabetes (**Figure 7**).

I feel like it’s sending me to an early grave. Just the chronic sleep deprivation of dealing with alarms overnight all the time, the decision fatigue, and everything. I probably shaved 15 years off my lifespan … We try to sleep seven to eight hours a night, but then we’re up four to six times a night on average.-3CG.

Families reported that frequent medical appointments, limited travel, and deviations from planned parenting approaches strained the family dynamic. Three parents spoke about siblings feeling excluded or believing the child with HI received special treatment; one parent described some psychological harm and feelings of inferiority in her other child. Interviewees also described the strain on parents’ interrelationships or marriage. In HIGR, 28% of caregivers reported negative effects, while 44% reported a strengthened relationship (**Table 6**).

Thankfully, my wife and I are a great team, and we’ve been able to lean on each other a lot. We also have our faith to draw on, but for those who don’t have those things, how do you find the resilience to do this on the daily without losing your mind?-10CG.

I think it probably strained our marriage a lot, because we were up. We were just tired. You’re just sleep deprived. We were both working full-time jobs and getting up all night long. The pressure—I know my husband felt a lot of pressure about, “I can’t change my job, because I’m her health insurance.”-6CG.

Financial considerations and access to healthcare and supplies generated additional stress. In HIGR, 52% of caregivers reported a “poor” or “fair” ability to afford costs associated with care (**Table 6**). Many families described changing careers, reducing work hours, or leaving the workforce. In HIGR, 20% of caregivers reported not working outside the home, and an additional 20% reported missing school/work “quite a lot” or “very frequently” in the last year. In some other cases, families described relying on in-home nurses or other caretakers for help with the child with HI. Two parents discussed comprehensive government programs providing social support, both during hospitalization and upon discharge, which allowed them to focus on caring for their child.

Two caregivers recalled journaling and three mentioned therapy as mechanisms to process their feelings. Caregivers expressed gratitude for support from patient organizations, particularly valuing family conferences and peer support as learning opportunities regarding disease management and connections with other families facing similar challenges (**Figure 7**).

I started to lose myself there. I became this other person that wasn’t tolerant to anything anymore because I felt like the only energy I have, I need to put into taking care of my son. If there is anything to add, it’s that I think without the therapy, we would not be feeling as good as we are today.-13CG.

When we went to the first conference, I cried … I was sobbing because I saw kids who were going through the same thing that my son was going through, and parents who were going through it too.-9CG.

### 3.11 Looking to the future

**Figure 8:**
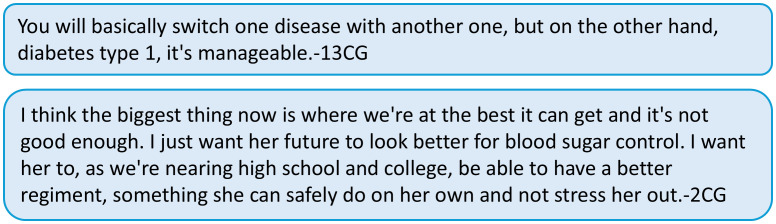
Reflections on current and future management. Additional quotes from the interviews related to current and future management post-pancreatectomy are presented. In the participant identifier, CG refers to the caregiver of someone with HI.

When asked to reflect on improvements in care that would benefit individuals with HI, interviewees discussed CGMs that are more reliable in the lower ranges, better adhesives, and greater knowledge about the natural history of the disease (**Figure 8**). Caregivers described the gaps in what was shared with them at the time of pancreatectomy, especially regarding the psychological anguish and medical trauma their child might experience.

I wish we had known the impact of all the medical trauma that he had as an infant, and that would affect him at almost 8 years old … a lot of it stems back to trauma, a scared baby that remembers physically being hurt, but doesn’t remember it in his brain, just like his body remembers it.-9CG.

Individuals wanted better treatment options, both for their child’s current care and to prevent surgery. Some caregivers acknowledged, however, regret that they did not advocate to remove more of their child’s pancreas, citing concern about avoiding brain damage with the current management options. Others believed that pancreatectomy was essential to avoid brain damage.

I don’t know how intense management would have been without that surgery, because I feel we could have put her at greater risk of other complications, more seizures or more permanent brain damage, more developmental delays. Anything like that would have been worse.-6CG.

Some people discussed concerns about disparities in places without access to critical HI tools and knowledge.

## 4 Discussion

This study investigated the lived experience of families who had a pancreatectomy for diffuse HI. While recent studies have begun incorporating patient voices in other areas of HI care, none have done so in the context of pancreatectomy (16, 17). Caregiver perspectives provide crucial insight into real-world decision-making, postoperative challenges, and long-term outcomes, all difficult to capture through clinical data alone. In this study, qualitative and quantitative findings were integrated across shared thematic areas. Together, these complementary data sources offer a more comprehensive understanding of the impacts of pancreatectomy than either approach alone.

Although pancreatectomy is not the first-line treatment for diffuse HI, it may be necessary when medical therapies fail to allow people with HI to go home from the hospital and avoid frequent, prolonged hypoglycemia (9). Families in this study faced this decision with critically ill children and limited alternatives. Ultimately, all caregivers interviewed said in the current treatment landscape, they would choose the surgery again, many believing it prevented irreversible brain injury and allowed children to go home from the hospital.

Subtotal pancreatectomy for diffuse HI is not curative; therefore, ongoing post-pancreatectomy management remained difficult for many families, which required similar on-going management as described in the literature (6, 11, 24, 25). All caregivers discussed ongoing challenges with glycemic stability, especially during exercise, highlighting an urgent need for more effective treatments to reduce the risks of hypoglycemia.

Individuals who believed prior to surgery that their child had focal HI only to learn intraoperatively the disease was diffuse, expressed anguish that highlights the importance of accurate presurgical diagnostics and clear conversations with parents experiencing extreme stress. Current imaging modalities such as ^18^F-DOPA PET are highly specialized, not universally available, and susceptible to misinterpretation without appropriate expertise (22, 31). Even with proper expertise, uncertainty can arise prior to surgical confirmation, suggesting a need for improved imaging technologies or interpretation methods to distinguish healthy tissue from HI-affected regions (31–33).

The COE model is anchored in adherence to clinical practice guidelines, multidisciplinary care, and external consultation to improve access and outcomes in rare diseases (19). Pancreatectomy surgery is complex and requires extensive expertise, and families should be able to access COE surgeons for this procedure. Some parents emphasized the importance of ongoing support, including collaboration between local clinicians and COE professionals. However, others desired clearer pathways for co-management arrangements. During stable periods of the HI journey, parents were more willing to receive care from local doctors. However, even parents whose children had transitioned to diabetes noted challenges with their child’s current management or perceived differences in their form of diabetes, though four of the six parents whose children had transitioned to diabetes stated that diabetes was easier to manage than ongoing hypoglycemia, which may have been due to the availability of a pump for insulin delivery. However, further research is needed to quantify these differences over time.

All parents described intense anxiety on the day of surgery. While some families expressed employment-related stressors during their child’s hospitalization, another family appreciated their country’s supportive parental leave policy, which reduced stress, improving their ability to cope. Adequate family-centered support programs should be implemented to support families in medical crises. Families in the study often described their gratitude for the HI specialist teams, yet felt that some general hospital employees could be more empathetic and utilize more compassionate communication strategies. Medical institutions can help provide services to lessen anxiety, including training staff regarding logistical and other support for parents facing long-term hospitalizations for their child (34, 35). Parents also described a desire to be heard; for example, a dialogue with clinicians around feeding produced better outcomes.

Feeding/eating issues were pervasive, often beginning before surgery and persisting for years. HI itself, medication side effects, and psychological stressors all contributed. Because feeding/eating is a critical component of HI management, some children with HI developed harmful associations with food that created challenging family dynamics. Future therapies should alleviate the need to use food as treatment.

Despite parents’ reported challenges associated with maintaining glycemic control and other disruptions to daily life, children did not report feeling different from their peers; even annoyances such as stopping activities due to lows were considered just part of their reality. This is likely due to the families’ dedication to building resilience within their children and on-going efforts to normalize their child’s life (18). Families reported finding support and strength in their faith, therapy, and connections with other families in the disease community.

Study participants shared the burden of living with hypoglycemia or diabetes post-pancreatectomy, including rigid feeding schedules, threats to glycemic stability, and the constant load of caregiving. These perspectives should inform care models, communication tools, and innovation. For instance, although CGMs are not FDA-approved for use in HI, many families rely on them to manage daily glucose monitoring. As research and clinical models evolve, integrating these lived experiences can guide more patient-centered innovations and policies that better reflect the complex reality of living with HI.

### 4.1 Strengths and limitations

A major strength of this study was the inclusion of unique qualitative data from interviews with people with HI and their caregivers after subtotal pancreatectomy alongside quantitative data from a registry. Moreover, the study was based on a global database, obtaining data from several countries. Lastly, our study was conducted in collaboration between a patient organization and healthcare professionals, leading to a shared prioritized focus on quality of life and alleviating family burden.

Some methodological limitations should be considered. This study had a relatively small sample size, which may restrict generalizability of our findings, especially when considering individual experiences summarized in interview quotes. The study relied heavily on self-reported data, including surgical history, hospitalizations, and clinical outcomes, which may be subject to recall bias and misclassification. In some cases, it was not possible to perform calculations within the whole HIGR dataset, particularly the median time from surgery to diabetes, due to categorical survey response options. In these cases, calculations were performed for the interview group, which also participated in the surveys.

Both the qualitative and quantitative components of this study relied on voluntary participation in HIGR. Recruitment for the study was conducted through CHI communication channels, which may have introduced selection bias toward those who are already engaged with CHI. This may limit representativeness, particularly for those with reduced access to patient organizations or limited resources, such as technology or internet connection. Further, while those who were interviewed were also included in the HIGR study, recruitment bias might have arisen for the interview participants, as interviews were conducted in English, limiting participation among non-English speaking individuals. However, this is less likely to have substantially impacted findings, as no significant differences were found between the interviewed and non-interviewed groups in their HIGR responses related to demographic information (**Table 1**), developmental delays, CGM use, frequency of hypoglycemia or hyperglycemia, presence of diabetes, or surgical complications.

This study integrates multiple data sources, including registry survey data, interview data, and CGM data, each with differing levels of completeness and structure. This heterogeneity may limit the direct comparability between data types. Additionally, the interpretation of long-term outcomes is constrained by the age distribution of the cohort, which includes young participants, including one interview participant less than two years of age at the time of data collection. As a result, outcomes such as post-pancreatectomy diabetes may not yet be fully observable in all participants.

Finally, the low wear time of CGM reflected in the data may impede assessment of glycemic trends or patterns in detail. In some cases, this was related to lack of insurance coverage for CGM devices among people with HI, particularly those without a diabetes diagnosis. Additionally, for participants who rely on a receiver to transmit CGM data, consistent data sharing is interrupted if the receiver is not synced with HIGR at least monthly. This may present a logistical burden for families navigating complex care needs. These challenges highlight the urgency for more equitable device access and streamlined data integration to support future research and clinical care in HI.

### 4.2 Future directions

Future investigations should seek to better understand post-pancreatectomy diabetes. This may include further evaluation of the way hypoglycemia is experienced and predicted in post-pancreatectomy diabetes compared to Type 1 diabetes using glycemic data from glucometers and CGM devices. Additionally, logistic regression analysis should be conducted to potentially identify predictors of post-pancreatectomy diabetes development. Therapeutic advancements are urgently needed for people with HI and should be considered for future studies. This includes new medications as well as supportive technology, such as a bi-hormonal pump developed for HI.

Additional exploration into themes raised during the interviews, including the persistence of feeding/eating issues and how they intersect with treatment modality and developmental stage should guide future studies. More research focused on emotional and psychosocial development in children and adolescents with complex medical needs could offer a strategy for long-term family support, and transition to adult care. There is also a need for more studies focused on pregnancy and efforts to achieve and maintain glycemic stability in women with HI. Additionally, more validation between patient and caregiver-reported data and clinical data will provide a more robust understanding of HI from multiple perspectives.

The findings from this study emphasize the importance of a patient- and family-centered approach to HI care and a need for better treatment options. While glycemic metrics are important, medical professionals should also consider the broader impacts of treatment decisions on quality of life, nutritional challenges, and family well-being. The need remains for improved educational resources and decision-support tools to clarify expectations for outcomes and follow-up care. Strengthening communication between families and care teams, fostering shared clinical decision-making, and ensuring that families have access to accurate, comprehensible information may improve confidence in treatment choices and promote better long-term satisfaction and engagement in care. Lastly, further consideration of the mental burden and anxiety surrounding this procedure affords an opportunity to provide compassionate care to families facing the decision to pursue pancreatectomy for diffuse HI.

## Data Availability

The raw data supporting the conclusions of this article will be made available by the authors, without undue reservation, upon reasonable request.

## Supplementary material

Supplementary Table 1 CGM data collected through HIGR.
