# Supplementary material for: The impact of subtotal pancreatectomy on people with congenital hyperinsulinism and their caregivers
Source: Front Endocrinol (Lausanne). 2026 Jun 22;17:1787064. doi: 10.3389/fendo.2026.1787064 (PMC13333396; doi:10.3389/fendo.2026.1787064)
Supplement: Supplementary Table 1 — CGM data collected through HIGR. [file Table1.docx]

# Supplementary Table 1: CGM data collected through HIGR

| **#** | **Data Range** | **Time Using CGM (Days)** | **Wear Time, Excluding Gaps (%)** | **Total Wear Time (%)** | **Avg Glucose (mg/dL)** | **CV (%)** | **TBR (%)** | **TIR (%)** | **TAR (%)** | **Clinical Information** | **Time Since Surgery** |
| --- | --- | --- | --- | --- | --- | --- | --- | --- | --- | --- | --- |
| 1 | All time | 1652 | 90.6 | 71.6 | 164 | 42.8 | 1.9 | 64.0 | 34.1 | Has diabetes | 10 years |
|  | 1 month | 31 | 97.8 | 97.8 | 145 | 33.3 | 0.8 | 75.5 | 23.7 |  |  |
| 2 | All time | 2163 | 70.9 | 46.0 | 185 | 40.0 | 2.0 | 51.9 | 46.0 | Has diabetes | 11 years |
|  | 1 month | 31 | 87.0 | 87.0 | 204 | 36.3 | 1.3 | 44.3 | 54.4 |  |  |
| 3 | All time | 2909 | 85.0 | 77.5 | 193 | 46.5 | 1.8 | 51.5 | 46.7 | Has diabetes | 11.5 years |
|  | 1 month | 31 | 91.0 | 91.0 | 291 | 34.6 | 1.4 | 15.7 | 82.8 |  |  |
| 4 | All time | 1609 | 95.0 | 94.6 | 176 | 41.0 | 1.3 | 59.3 | 39.4 | Has diabetes | 6-7 years |
|  | 1 month | 31 | 95.7 | 95.7 | 161 | 44.5 | 1.8 | 67.2 | 31.0 |  |  |
| 5 | All time | 1424 | 96.2 | 88.9 | 171 | 28.2 | 0.4 | 63.0 | 36.6 | Has diabetes | 29 years |
|  | 1 month | 31 | 96.9 | 96.9 | 161 | 28.1 | 0.3 | 72.2 | 27.5 |  |  |
| 6 | All time | 1975 | 94.9 | 81.9 | 143 | 40.8 | 6.9 | 69.4 | 23.7 | Has diabetes | 7 years |
|  | 1 month | 23 | 80.9 | 64.1 | 207 | 34.9 | 0.8 | 41.5 | 57.7 |  |  |
| 7 | All time | 946 | 77.7 | 36.3 | 193 | 33.8 | 0.5 | 46.1 | 53.5 | Has diabetes | 11 years |
|  | 1 month^a^ | 9 | 83.2 | 24.1 | 179 | 46.9 | 0.1 | 52.4 | 47.4 |  |  |
| 8 | All time^b^ | 385 | 85.2 | 73.2 | 168 | 28.3 | 0.3 | 66.0 | 33.7 | Has diabetes | 22 years |
| 9 | All time | 21 | 73.6 | 17.0 | 171 | 38.6 | 1.4 | 62.3 | 36.3 | No medication, no diabetes | 14 years |
|  | 1 month | 2 | 46.4 | 46.4 | 202 | 33.5 | 0.0 | 48.7 | 51.3 |  |  |
| 10 | All time^b^ | 1740 | 78.6 | 49.0 | 191 | 35.3 | 0.9 | 48.3 | 50.9 | No medication, no diabetes | 14 years |
| 11 | All time | 534 | 86.7 | 54.7 | 125 | 31.6 | 4.6 | 87.1 | 8.4 | No medication, uses G-tube, no diabetes | 2 years |
|  | 1 month | 16 | 77.1 | 64.9 | 139 | 38.6 | 5.9 | 72.8 | 21.3 |  |  |
| 12 | All time | 529 | 96.1 | 95.6 | 107 | 32.6 | 11.3 | 85.3 | 3.4 | Taking long-acting octreotide, uses G-tube, no diabetes | 1.5 years |
|  | 1 month^a^ | 22 | 97.3 | 69.0 | 105 | 32.8 | 14.5 | 82.8 | 2.6 |  |  |
| 13 | All time | 72 | 66.2 | 7.1 | 145 | 36.8 | 2.2 | 77.6 | 20.2 | Taking lanreotide, no diabetes | 9 years |

^a^ Data from the last month was not available; therefore, data was reported from within 1 month of the latest submission of the *Glucose Monitoring* survey in HIGR.

CV= Coefficient of variation; TBR= Time below range; TIR= Time in range; TAR= Time above range

^b^ Participants that have not shared data from within the last 18 months; therefore, 1 month data would not be representative of recent time.
